# Evidence-based clinical standard for the diagnosis and treatment of candidemia in critically ill patients in the intensive care unit

**DOI:** 10.1016/j.bjid.2024.104495

**Published:** 2024-12-21

**Authors:** Jorge Alberto Cortés, Martha Carolina Valderrama-Rios, Paula M. Peçanha-Pietrobom, Moacyr Silva Júnior, Cándida Diaz-Brochero, Rafael Ricardo Robles-Torres, Carmelo José Espinosa-Almanza, Laura Cristina Nocua-Báez, Marcio Nucci, Carlos Arturo Álvarez-Moreno, Flavio Queiroz-Telles, Ricardo Rabagliati, Rita Rojas-Fermín, Jorge L. Finquelievich, Fernando Riera, Patricia Cornejo-Juárez, Dora E. Corzo-León, Luis E. Cuéllar, Jeannete Zurita, Augusto Raúl Hernández, Arnaldo Lopes Colombo

**Affiliations:** a Universidad Nacional de Colombia, Facultad de Medicina, Departamento de Medicina Interna, Bogotá, Colombia; b Hospital Universitario Nacional de Colombia, Unidad de Infectología, Bogotá, Colombia; c Universidade Federal de São Paulo, Escola Paulista de Medicina, Departamento de Medicina, Divisão de Enfermidades Infecciosas, São Paulo, SP, Brasil; d Hospital Israelita Albert Einstein, São Paulo, SP, Brasil; e Pontificia Universidad Javeriana, Hospital Universitario San Ignacio, Departamento de Medicina Interna, Bogotá, Colombia; f Universidade Federal do Rio de Janeiro, Hospital Universitário, Departament of Internal Medicine, Rio de Janeiro, RJ, Brazil; Grupo Oncoclínicas, Brazil; g Clínica Universitaria Colombia, Clínica Colsanitas Grupo Keralty, Bogotá, Colombia; h Universidade Federal de Paraná, Hospital de Clínicas, Departamento de Saúde Pública, Curitiba, PR, Brasil; i Pontificia Universidad Católica de Chile, Escuela de Medicina, Department of Adult Infectious Diseases, Santiago, Chile; j Hospital General Plaza de la Salud, Santo Domingo, República Dominicana; k Universidad de Buenos Aires, Facultad de Medicina, Centro de Micología, Buenos Aires, Argentina; l División de Enfermedades Infecciosas, Sanatorio Allende Córdoba, Córdoba, Argentina; m Universidad Nacional de Córdoba, Enfermedades Infecciosas, Córdoba, Argentina; n Instituto Nacional de Cancerología, Departamento de Infectología, Ciudad de México, Mexico; o Universidad de Exeter, Centro de Micología Médica del Medical Research Council, Exeter, Reino Unido; p Instituto Nacional de Enfermedades Neoplásicas, Lima, Perú; q Pontificia Universidad Católica del Ecuador, Facultad de Medicina, Quito, Ecuador; r Zurita & Zurita Laboratorios, Unidad de Investigaciones en Biomedicina, Quito, Ecuador; s Hospital Pacifica Salud, Departamento de Medicina Crítica, Ciudad de Panamá, Panamá; t Antimicrobial Resistance Institute of São Paulo (ARIES), São Paulo, SP, Brasil

**Keywords:** Candidemia, Critical illness, Intensive care units, Antifungals, Latin America

## Abstract

Candidemia is the predominant form of invasive candidiasis and the most frequently occurring serious fungal infection in critically ill patients in Intensive Care Units (ICU). Studies carried out in Latin America reveal a higher incidence of candidemia and higher mortality rates when compared to North America or Europe. This highlights the need to develop guidelines for correctly diagnosing and treating candidemia in critically ill patients in the ICU. These guidelines are part of the efforts to implement antifungal optimization programs in the region to obtain better clinical outcomes and promote rational antifungal use. This evidence-based clinical standard, established through expert consensus for the Latin American context, contains recommendations and algorithms for diagnosing and treating candidemia in critically ill ICU patients.

## Introduction

Candidemia is a fungal infection of the bloodstream caused by a member of the *Candida* species.^1^ It is the most common form of invasive candidiasis, which can originate endogenously due to rupture of barriers in the gastrointestinal tract, the endogenous reservoir of these yeasts, or exogenously as a result of iatrogenic contamination of intravascular catheters during health care.^1^, ^2^, ^3^, ^4^ Candidemia is the most frequent severe fungal infection in critically ill patients in the Intensive Care Unit (ICU).^3^^,^^5^ The incidence of candidemia in this population may vary according to factors such as local epidemiology, patient conditions, medical care practices, and outbreak occurrence.^2^^,^^6^, ^7^, ^8^, ^9^

The availability of appropriate and accurate diagnostic tests is crucial to producing data on the frequency of presentation of fungal infections, and in many countries, fungal infections are not mandatory reporting. Thus, the estimates available from a few population-based studies may underestimate the frequency of these diseases worldwide.^10^ However, authors around the world have tried to describe the disease's frequency in their countries, finding widely varying data,^10^ with Latin America exhibiting a higher candidemia incidence than North America or Europe and a constant tendency to increase in recent years.^11^, ^12^, ^13^, ^14^, ^15^ In the last two decades, there has been a change in the distribution of *Candida* species, with fewer bloodstream infections by *Candida albicans*, and more infections by non-albican *Candida* species,^2^^,^^16^, ^17^, ^18^, ^19^, ^20^ with consequences in the use of antifungals in daily clinical practice, due to the lower susceptibility of *C. parapsilosis, Nakaseomyces glabratus* (*Candida glabrata*), and *C. auris* to different antifungals, especially to azoles.^2^^,^^19^^,^^21^ This could negatively impact candidemia-related hospital morbidity and mortality associated with candidemia.^5^^,^^22^

The frequency of antifungal resistance is increasing worldwide.^23^ A reflection of this problem is the recent World Health Organization (WHO) publication of critical, high- and medium-priority fungal pathogens to guide research, development, and public health actions, which include numerous *Candida* species: *Candida auris, Candida albicans, Nakaseomyces glabratus* (*Candida glabrata*), *Candida tropicalis, Candida parapsilosis,* and *Pichia kudriavzevii* (*Candida krusei*).^24^ Additionally, the complexity and recent changes in the taxonomy of what were previously known as *Candida* species can make the problem difficult to appreciate and limit the clinical action of clinicians unfamiliar with the names. Hence, a summary of the major nomenclatural changes in clinically important *Candida* species is presented in Table 1.

**Table 1:** Nomenclature changes in *Candida* species of clinical importance.

Previous name	Current name
*Candida bracarensis*	*Nakaseomyces bracarensis*
*Candida catenulata*	*Diutina catenulata*
*Candida colliculosa*	*Torulaspora delbrueckii*
*Candida eremophila*	*Pichia eremophila*
*Candida etchellsii*	*Starmerella etchellsii*
*Candida fabianii*	*Cyberlindnera fabianii*
*Candida famata*	*Debaryomyces hansenii*
*Candida fermentati*	*Meyerozyma caribbica*
*Candida glabrata*	*Nakaseomyces glabrata*
*Candida guilliermondii*	*Meyerozyma guilliermondii*
*Candida inconspicua*	*Pichia cactophila*
*Candida kefyr, Candida pseudotropicalis*	*Kluyveromyces marxianus*
*Candida krusei*	*Pichia kudriavzevii*
*Candida lambica*	*Pichia fermentans*
*Candida lipolytica*	*Yarrowia lipolytica*
*Candida lusitaniae*	*Clavispora lusitaniae*
*Candida nivariensis*	*Nakaseomyces nivariensis*
*Candida neorugosa*	*Diutina neorugosa*
*Candida norvegensis*	*Pichia norvegensis*
*Candida pararugosa*	*Diutina pararugosa*
*Candida pelliculosa, Pichia anomala*	*Wickerhamomyces anomalus*
*Candida pintolopesii*	*Kazachstania telluris*
*Candida pseudorugosa*	*Diutina pseudorugosa*
*Candida pulcherrima*	*Metschnikowia pulcherrima*
*Candida rugosa*	*Diutina rugosa*
*Candida sorbosivorans*	*Starmerella sorbosivorans*
*Candida utilis*	*Cyberlindnera jadini*

Taken and adapted from Kidd SE, et al. Open Forum Infect Dis, 2023.^25^ Borman AM, et al. J Clin Microbiol, 2021.^26^.

Given the aforementioned aspects, recommendations and algorithms are needed to guide daily clinical practice in diagnosing and treating candidemia in critically ill ICU patients. This, as part of Latin America's need for the implementation of antifungal optimization programs,^11^ to enhance health care, clinical outcomes, and antifungal use.

### Scope

This Evidence-Based Clinical Standard (EBCS) is intended for healthcare personnel involved in caring for critically ill adult ICU patients (over 18 years of age) and for decision-makers or entities involved in planning health policies in healthcare institutions in Latin America.

### Target

To develop recommendations and algorithms for candidemia diagnosis and treatment in critically ill adult ICU patients systematically and collaboratively using the best available evidence.

### Patients considered and clinical aspects addressed

The clinical practice recommendations and algorithms contained in this EBCS are aimed at critically ill adult ICU patients (equal to or older than 18 years of age) with a clinically suspected or confirmed diagnosis of candidemia and include interventions for diagnosis and treatment.

### Patients not considered and clinical aspects not addressed

This EBCS excludes the following patient groups: children or adolescents (under 18 years of age), pregnant women, patients living with human immunodeficiency virus, solid organ or hematopoietic progenitor transplant recipients, or patients with neutropenia. The EBCS does not cover strategies for candidemia prophylaxis in critically ill patients hospitalized in the ICU.

### Users to whom the clinical practice guide is directed and healthcare field

The clinical practice recommendations and algorithms contained in this EBCS are aimed at health professionals involved in the care of critically ill adult ICU patients, including physicians specializing in internal medicine, critical medicine and intensive care, infectious diseases, nurses, pharmaceutical chemists, clinical laboratory personnel, and other personnel involved in the care process of this demographic.

## Methodology

The clinical practice recommendations and algorithms contained in this EBCS have been developed through a process proposed by the National University Hospital of Colombia, in collaboration with the National University of Colombia and the Clinical Research Institute of the National University of Colombia, called Evidence-Based Clinical Standards,^27^ consists of six sequential phases: 1) EBCS development group composition; 2) Definition EBCS´s scope and objective; 3) Systematic search for clinical practice guidelines (CPG); 4) Screening, quality evaluation, and selection of CPG; 5) Elaboration of preliminary recommendations and algorithms; 5a) Elaboration of a comparative table of evidence; 5b) Review and discussion of recommendations and algorithms by the development group; 6) Final elaboration of recommendations and algorithms; 6a) Review and discussion of recommendations and algorithms in a participatory process with expert consensus.

### EBCS development group composition

The EBCS development group comprised eight members, including thematic and methodological experts. These experts included physicians specializing in internal medicine, infectious diseases, critical medicine and intensive care, and clinical epidemiology, with experience in systematic literature reviews, the synthesis and qualification of evidence, and participatory processes (JAC, MCV, PMMP, MSJ, CJEA, CDB, RRR, and LCN). Prior to commencing activities, each member of the development group made the conflict-of-interest declaration by filling out the designated form. In instances where a conflict was declared, analysis was conducted to determine its implications for participation.

### Definition of scope and objective

The final formulation of the scope, objective, patient population to whom the recommendations and algorithms will be applied, clinical aspects to be addressed, and user population and care setting to which the content of the EBCS is addressed was guided by tracer questions: why is it being done?, is there variability in current practice?, what is it being done for?, who is it intended for?, and who will use it?^28^

### Systematic search of CPG

Systematic searches were conducted to identify CPGs that corresponded to the proposed scope and objective and were published between 2014 and 2023, without regard for language.

Highly sensitive electronic search strategies were designed. The search was conducted between June 12 and 15, 2023, on the websites of the following CPG compiling and developing entities: Guidelines International Network, Agency for Healthcare Research and Quality/National Guidelines Clearinghouse (AHRQ), CMA Infobase: Clinical Practice Guidelines, Catalogue of Clinical Practice Guidelines in the National Health System, National Institute for Clinical Excellence, Scottish Intercollegiate Guidelines Network and WHO. Additionally, the Medline and Embase databases were searched by means of strategies customized for each search engine. These strategies included boolean, truncation, and proximity operators, free text terms, and controlled vocabulary using key terms such as “candida”, “candidemia”, “intensive care unit”, and “critical illness”. More information on the search strategies is presented in the Supplementary Material in Table 1.

### Screening, quality assessment and selection of CPGs

Upon obtaining the systematic search results, two reviewers (CDB, LCN) independently screened and selected the primary references by title and abstract. They selected the references that corresponded to CPGs, expert consensus, or the generation of recommendations that addressed the aspects defined in this guide's scope and objective. Subsequently, two reviewers (CDB, LCN) independently conducted the screening and secondary selection in full text of the references selected in the previous step. They used the criteria outlined in the modified tool 7 of the Methodological Guide for the adoption - adaptation of evidence-based clinical practice guidelines of the Ministry of Health and Social Protection of Colombia^29^: CPG with the generation of evidence-based recommendations, CPG with a development process and the conformation of a developer group, CPG with a reliable evidence search, the date of the last update of the search, and the use of the GRADE (Grading of Recommendations, Assessment, Development, and Evaluation) system for the global grading of evidence (Table 2). References without access to the full text were excluded. Discrepancies between the two reviewers were resolved by a process of review, discussion, and consensus, or with the involvement of a third reviewer.

**Table 2:** Overall grading of evidence using the GRADE system.

Levels of certainty of evidence^33^	
High	There is high confidence that the true effect is close to the effect estimate.
Moderate	The confidence in the effect estimate is moderate. The true effect may be close to the estimate, but it could be substantially different.
Download	Confidence in the effect estimate is limited. The true effect may be substantially different from the effect estimate.
Very low	The confidence in the effect estimate is very low. The true effect is likely to be substantially different from the effect estimate.
**Meaning of the strength and direction of the recommendations**^33^	
Strongly in favor	The benefits of the intervention clearly outweigh the undesirable effects.
Conditionally in favor	The benefits of the intervention probably outweigh the undesirable effects.
Strongly against	The undesirable effects of the intervention clearly outweigh the benefits.
Conditionally against	The undesirable effects of the intervention probably outweigh the benefits.
**Implications of the strength of the recommendation**^34^	
The implications of a strong recommendation are	
For patients	Most people in this situation would desire the recommended course of action, and only a small proportion would not.
For clinicians	Most patients should receive the recommended course of action.
For policy makers	The recommendation can be adopted as policy in most situations.
The implications of a conditional recommendation are	
For patients	Most people in this situation would like the recommended course of action, but many do not.
For clinicians	You should recognize that different options will be appropriate for different patients, and you should help each patient arrive at a management decision consistent with his or her values and preferences.
For policy makers	Policy formulation will require substantial debate and the involvement of many stakeholders

The CPGs selected after the screening described above were submitted to the development group for quality assessment using the AGREE II tool.^30^ Three reviewers, including a clinical expert and a methodological expert, independently evaluated each guideline. In cases where CPG evaluation required additional information, a request for complementary information was sent to the developer groups via email. The CPGs with a compliance rate of 60 % or higher in the domains of methodological rigor and editorial independence were identified and selected as a consequence of the quality assessment process.

With the methodology described, two references were selected: “Clinical Practice Guideline for the Management of Candidiasis: 2016 Update by the Infectious Diseases Society of America”, published in 2016,^31^ and “Colombian consensus on the diagnosis, treatment, and prevention of *Candida* spp. disease in children and adults”, published in 2019.^32^ The process of screening and selection of CPGs is summarized in the PRISMA diagram (Supplementary Material Fig. 1).

### Preparation of proposed recommendations and preliminary algorithm

To develop the preliminary recommendations and algorithms, a comparative table was created based on the review and extraction of information from the two selected CPGs. This table included the recommendations identified in each of the selected references for each of the previously defined clinical aspects to be addressed in the EBCS, as well as the respective level of certainty of the evidence and strength of the recommendation. All the members of the development group participated in informal virtual consensus meetings that lasted approximately 2 hours each. During these meetings, the information extracted from the comparative table was presented, reviewed, and discussed, and the preliminary recommendations and algorithms of the EBCS were constructed.

### Expert consensus

Finally, the proposal of recommendations and the preliminary algorithm was taken to an expert consensus in which professionals from Argentina (FR, JF), Brazil (PMPR, MSJ, MN, FQT, ALC), Chile (RR), Colombia (JAC, CDB, RRR, CJEA, LCN, CAA), Ecuador (JZ), Mexico (PCJ, DEC), Panama (ARH), Peru (LEC), and the Dominican Republic (RRF), with training in infectious diseases (JAC, PMP, ALC, MN, CAA, FQT, RR, RR, JLF, FR, PCJ, DEC, LEC, LCN), microbiology (JZ), critical medicine and intensive care (MSJ, CJEA, ARH), and internal medicine (CDB, RRR), thus including the perspective of different services or care areas involved in the processes of diagnosis and treatment of candidemia in critically ill adult patients hospitalized in the ICU in Latin America. Each participant in the consensus declared their conflicts of interest by filling out the designated form. In cases where a conflict-of-interest was declared, analysis was carried out to determine its implications for participation.

The preliminary algorithms and recommendations were presented and reviewed during two virtual consensus meetings, each lasting approximately four hours. The final algorithm and recommendations were formulated and constructed through a participatory process that was conducted in real time using Delphi methodology. Voting was carried out anonymously, by electronic means, to evaluate the degree of agreement with each recommendation and section of the algorithm using a Likert-type scale with scores ranging from 1 to 9, where 1 corresponded to strongly disagree, 5 to neither agree nor disagree, and 9 to completely agree.^35^ Agreement was defined as the presence of ≥ 60 % of the votes in the scale range of 4 to 9 and < 20 % of the votes in the 4 to 6 range of the scale. Conversely, there was no agreement (i.e., no consensus) when ≥40 % of the votes were in the range of 1 to 3 on the scale. In cases in where there was no agreement in the first round, a discussion session and a new round of voting were held. Each query was permitted to have a maximum number of three rounds.

## Results

### Flowchart

**Fig. 1:**
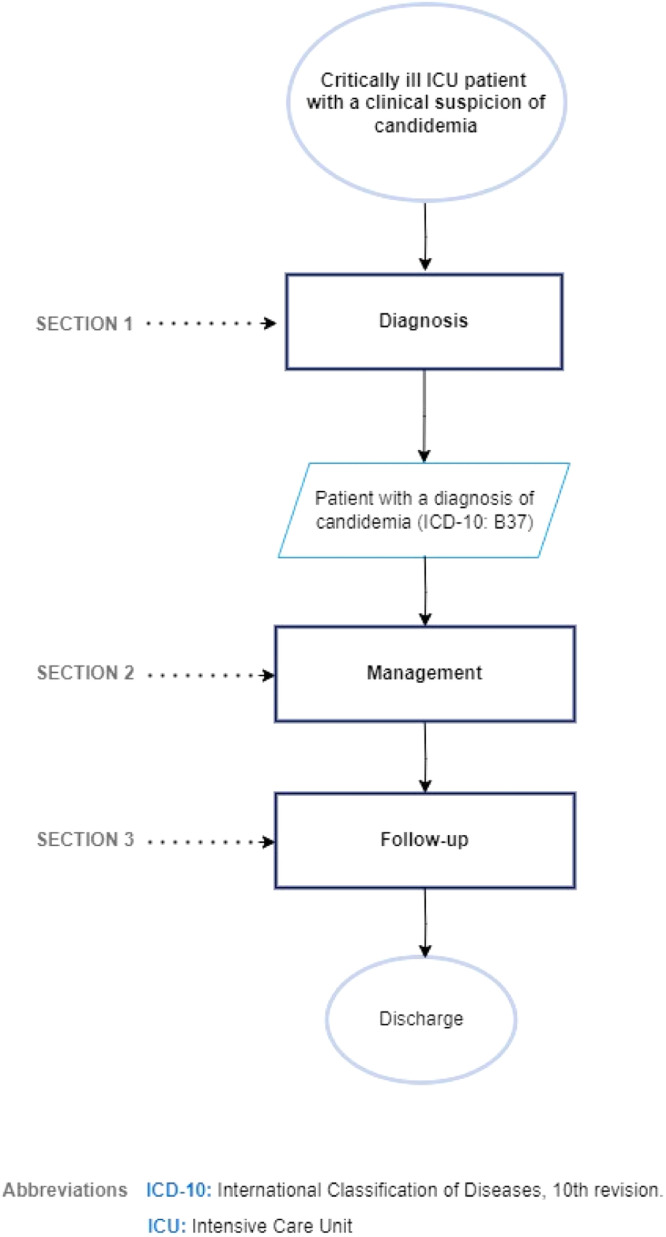
Flow chart for the diagnosis and treatment of candidemia in critically ill ICU patients. ICD-10, International Classification of Diseases, 10th revision; ICU, Intensive Care Unit.

Fig. 1 illustrates the flow chart for the diagnosis and treatment of candidemia in critically ill ICU patients.

**Fig. 2:**
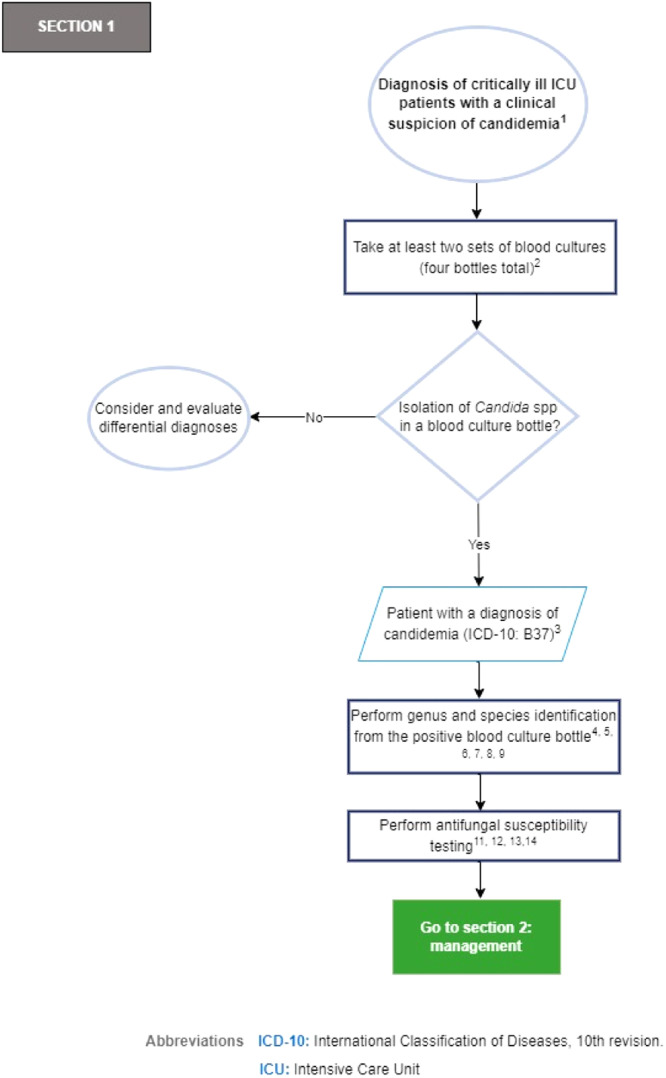
Section 1 of the flowchart: Diagnosis of critically ill ICU patients with a clinical suspicion of candidemia. ICD-10, International Classification of Diseases, 10th revision; ICU, Intensive Care Unit.

In Fig. 2, Section 1 of the flowchart (diagnostic approach to the critically ill patient in the ICU with clinical suspicion of candidemia) is presented.

Recommendations flowchart, Section 1: Diagnosis of critically ill ICU patients with a clinical suspicion of candidemia.

1. It is recommended to evaluate the risk of candidemia in critically ill ICU patients with sepsis without a clinically identified focus (expert recommendation).^5^^,^^36^
2. Two sets of blood cultures (four bottles in total): In critically ill ICU patients with suspected candidemia, it is recommended that at least two sets of blood cultures (four bottles in total) be obtained sequentially from different sites (expert recommendation).^37^ To ensure optimal isolation of microorganisms, it is essential to follow the blood sampling and processing guidelines of the health care institution where the procedure is performed.^37^
3. The presence of a *Candida* spp. isolate in a single blood culture bottle from peripheral blood or blood drawn through a Central Venous Catheter (CVC) is considered proven candidemia (strong recommendation, high quality evidence, GRADE).^32^
4. It is recommended to perform Gram staining of the positive blood culture sample and inform the treating clinical group (strong recommendation, high quality evidence, GRADE).^32^
5. It is recommended to perform genus and species identification in mycological culture in cases of proven candidemia (strong recommendation, high quality evidence, GRADE).^32^
6. It is recommended to prefer genus and species identification methods by proteomics (MALDI-TOF MS, Matrix-Assisted Laser Desorption/Ionization Time-Of-Light Mass Spectrometry) over automated or semi-automated identification methods with the latest update of libraries, according to local availability (strong recommendation, high quality evidence, GRADE).^32^^,^^38^ Some of the advantages and disadvantages of *Candida* spp. identification methods in patients with fungemia to be considered are presented in Table 3.

**Table 3:** Advantages and disadvantages of the methods for identification of *Candida* spp. in patients with fungemia.

Identification methods	Advantages	Disadvantages
Conventional; culture in microbiological media	It can quickly guide the diagnosis (visualization of yeast-like structures).	Long process (24–72 h)
	Economic	Low sensitivity
	Allows for antifungal susceptibility testing	
	Widely available	
	Extensive experience	
Automated and semi- automated methods	Faster, more sensitive and require less manual labor than conventional methods	Less rapid and accurate than methods based on nucleic acid amplification or proteomics.
	Economic	
	Widely available	
	Extensive experience	
Nucleic acid amplification (PCR)-based methods	Very fast results (hours)	Mostly developed “in house”, with limited clinical validation
	Sensitive, specific, and precise	Performed only in reference laboratories, which limits the advantage of short detection time
	Extensive clinical validation for the detection of Candida species.	The heterogeneity of the tests makes it difficult to interpret the data
		Need for trained laboratory personnel to perform the test
		Not universally available
Mass Spectrometry (MALDI-TOF)	Very fast results (minutes)	High initial cost of MALDI- TOF equipment
	Sensitive, specific, and precise	Not universally available
	Identification of microbial pathogens directly from positive blood cultures	Requires positive blood culture
	Less expensive than molecular and immunological methods.	

Taken and adapted from Singhal N, et al. Front Microbiol, 2015.^47^ Soriano A, et al. J Antimicrob Chemother, 2023.^43^ Dingle TC, et al. Clin Lab Med, 2013.^48^.
7. It is recommended that automated or semi-automated identification methods with the latest library update be preferred over manual identification methods (strong recommendation, high quality evidence, GRADE)^32^ (Table 3).
8. In the event of the availability of PCR for detection of *Candida* spp. or MALDI-TOF MS, it is recommended to perform the procedure on a sample of the positive blood culture bottle of the yeast fungus (strong recommendation, moderate quality evidence, GRADE)^32^^,^^38^^,^^39^ (Table 3).
9. If MALDI-TOF MS is available, it is recommended to perform this test on the colonies identified for the detection of *Candida* spp. species (expert recommendation)^38^^,^^40^^,^^41^ (Table 3).
10. In the event of the availability of serum biomarkers such as 1,3-β-d-glucan, consider their use as a complementary diagnostic and prognostic tool (weak recommendation, moderate quality evidence, GRADE).^32^
11. Azole susceptibility testing is recommended for all *Candida spp*. isolates from the bloodstream (strong recommendation, low quality evidence, GRADE).^31^
12. Echinocandin susceptibility testing is recommended in patients who have received prior treatment with an echinocandin and in those with *N. glabratus* (*C. glabrata*) or *C. auris* infection (strong recommendation, low quality evidence, GRADE).^31^^,^^42^^,^^43^
13. Consider performing echinocandin susceptibility testing for all bloodstream isolates of *Candida* spp. for epidemiological purposes (expert recommendation).^44^
14. It is recommended to perform antifungal susceptibility testing by broth microdilution (commercial or manual, according to the methodology approved by the European Committee on Antimicrobial Susceptibility Testing [EUCAST] or the Clinical Laboratory Standards Institute [CLSI]) or concentration gradient strips (expert recommendation).^45^^,^^46^ To guarantee an adequate interpretation of the antifungal susceptibility tests, it is essential to work in coordination with the microbiology service of the health care institution where the procedure is carried out to ensure that the final points generated for each species reflect those of the reference methods.^37^

**Fig. 3:**
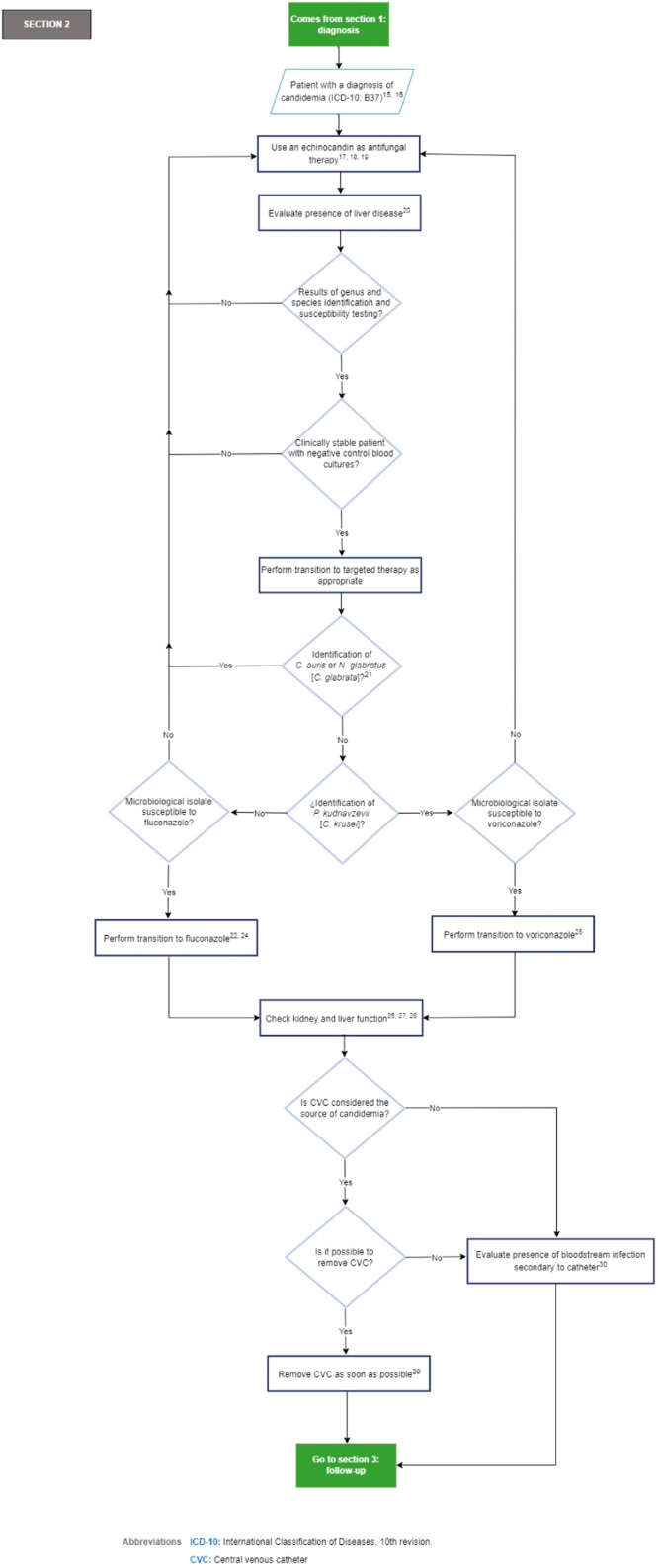
Section 2 of the flowchart: Management of critically ill ICU patients with a diagnosis of candidemia. ICD-10, International Classification of Diseases, 10th revision; ICU, Intensive Care Unit.

Fig. 3 depicts section 2 of the flowchart (treatment of the critically ill ICU patients with a diagnosis of candidemia).

Recommendations section 2 of the flowchart: Management of the critically ill ICU patients with a diagnosis of candidemia.

15. It is recommended to treat all patients who have a positive blood culture for a yeast species immediately (expert recommendation).^5^^,^^49^
16. The specialist panel acknowledges that further studies are needed to evaluate the characteristics of patients who benefit from empiric therapy. Good practice point: the routine initiation of empiric antifungal therapy is discouraged in low-risk patients according to risk scores (i.e., Candida Colonization Index [CCI], Candida score, EORTC/MSG [European Organization for Research and Treatment of Cancer/Invasive Fungal Infections Cooperative Group and the Mycoses Study Group Education and Research Consortium]).^50^^,51^
17. An echinocandin (caspofungin [70 mg loading dose, then 50 mg per day IV]; micafungin [100 mg per day]; anidulafungin [200 mg loading dose, then 100 mg per day]) is recommended as antifungal therapy (strong recommendation, high quality evidence, GRADE).^31^
18. There are no differences between echinocandins in the clinical setting of non-neutropenic patients. The choice will depend on drug interactions, hepatic impairment, side effects, and treatment costs (Strong recommendation, moderate quality evidence, GRADE).^32^
19. In the setting of intolerance, resistance, or therapeutic failure to echinocandins, it is recommended to use lipid formulations of amphotericin B (liposomal amphotericin B [3 mg/kg day], lipid complex amphotericin B [5 mg/kg day]) over the use of deoxycholate amphotericin B (0.7–1 mg/kg day), as an acceptable alternative antifungal therapy (strong recommendation, moderate quality evidence, GRADE).^32^
20. Echinocandins, preferably anidulafungin (200 mg loading dose, then 100 mg daily), are recommended as antifungal therapy for candidemia in patients with Child-Pugh B or C class liver failure (expert recommendation).[52]
21. The expert panel considers that further studies are needed to evaluate the appropriateness of transitioning to higher doses of fluconazole (800 mg [12 mg/kg] daily) or voriconazole (200 to 300 mg [3 to 4 mg/kg] twice daily) in the setting of N. glabratus (*C. glabrata*) candidemia when the microbiologic isolates are susceptible to fluconazole and/or voriconazole.
22. In patients who are clinically stable, have isolates that are susceptible to fluconazole, have negative control blood cultures, are endocarditis-free, and have undergone CVC removal when the CVC is considered the source of infection, it is recommended to transition from an echinocandin to fluconazole. This transition should be initiated with a loading dose of 800 mg (12 mg/kg), followed by 400 mg per day (6 mg/kg) over a period of 5 to 7 days. The route of administration should be determined by the clinical condition and tolerance of the patient (strong recommendation, moderate quality evidence, GRADE).^31^^,^^43^^,53^
23. Transition from amphotericin B to fluconazole after 5 to 7 days (IV or oral according to clinical condition and tolerance) is recommended in patients who are clinically stable, with isolates that are susceptible to fluconazole, with negative control blood cultures, and without endocarditis (strong recommendation, high quality evidence, GRADE).^31^^,^^43^
24. It is recommended that voriconazole (400 mg twice daily in 2 doses, then 200 mg twice daily) be considered as an alternative in patients with isolation of non-albicans *Candida* (except for *N. glabratus* [*C. glabrata*] or *C. auris*) (expert opinion, low quality evidence, GRADE).^31^^,54^
25. Voriconazole is recommended as an oral de-escalation therapy for selected cases of P. kudriavzevii (*C. krusei*) candidemia (strong recommendation, low quality evidence, GRADE).^31^
26. In patients with renal insufficiency and candidemia receiving fluconazole for antifungal treatment, it is recommended that doses be adjusted according to the creatinine clearance value 48 hours after commencing antifungal treatment (strong recommendation, high quality evidence, GRADE).^32^^,55^
27. Good practice point: Avoid intravenous voriconazole in patients with renal insufficiency with creatinine clearance less than 50 mL/min.^31^
28. Caution and consideration of hepatotoxicity of voriconazole is recommended in patients with Child-Pugh B or C class liver failure (expert recommendation).^31^
29. The CVC should be removed as early as possible during candidemia when the source of infection is presumed to be the CVC and the CVC can be safely removed, even though the CVC is not the source of candidemia in most patients. The decision to remove the CVC should be customized to the specific needs of each patient (strong recommendation, moderate quality evidence, GRADE).^31^
30. Good practice point: When the CVC cannot be removed, obtain cultures through the CVC and peripheral venous puncture.[^56^]

**Fig. 4:**
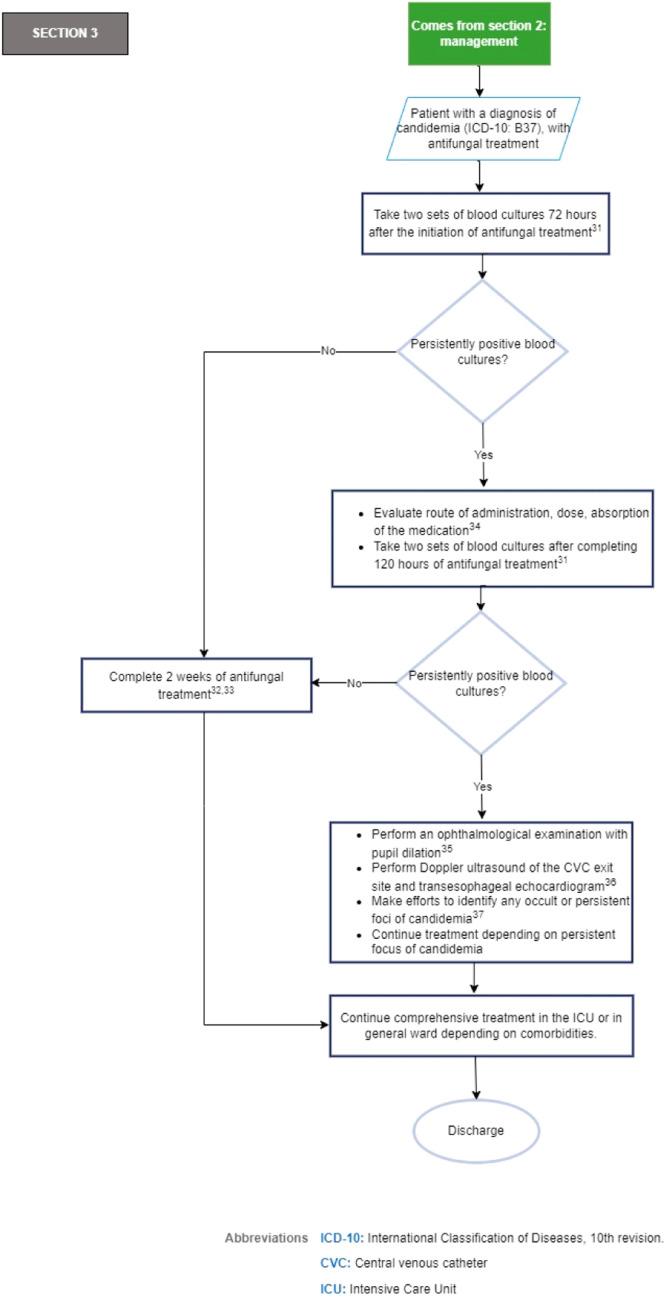
Section 3 of the flowchart: Follow-up of the critically ill ICU patients with treatment for candidemia. ICD-10, International Classification of Diseases, 10th revision; CVC, Central Venous Catheter; ICU, Intensive Care Unit.

Fig. 4 shows section 3 of the flowchart (follow-up of the critically ill ICU patients who received treatment for candidemia).

Recommendations section 3 of the flowchart: Follow-up of the critically ill ICU patients with treatment for candidemia.

31. To establish the time at which candidemia has been eliminated, it is recommended two sets of follow-up blood cultures be obtained three days after the initiation of treatment and at five days if candidemia persists (strong recommendation, low quality evidence, GRADE).^31^^,57^
32. Until there are randomized comparative studies evaluating shorter durations of antifungal therapy in patients with candidemia, the recommended duration of treatment for candidemia without obvious metastatic complications is two weeks after the documented clearance of *Candida spp*. from the bloodstream and the resolution of symptoms attributable to candidemia (strong recommendation, moderate quality evidence, GRADE).^31^
33. In the event of the availability of serum biomarkers such as 1,3-β-d-glucan, consider their use for the suspension of empirical therapy or as a tool for the analysis of prognostic factors (weak recommendation, moderate quality evidence, GRADE).^32^
34. Good practice point: In patients with persistently positive blood cultures, it is suggested to review the CVC if it has not been removed, as well as the route of administration, dose, and absorption of the antifungal treatment established.[^58^]
35. Consider ophthalmologic evaluation with pupil dilation after diagnosis in patients with symptoms or signs of decreased visual acuity, patients under deep sedation or altered consciousness, or those with persistent candidemia (expert recommendation).[^59^]
36. If blood cultures are persistently positive five days after the initiation of antifungal therapy, if there are clinical signs indicative of endocarditis, or in patients with risk factors for endocarditis*, Doppler ultrasound of the CVC exit site and transesophageal echocardiogram are recommended (strong recommendation, low quality evidence, GRADE).^32^ *Risk factors for endocarditis such as valvular heart disease, use of valvular prostheses, previous cardiac surgery, and long-term catheters.[^60,61^]
37. Good practice point: In patients with persistent candidemia five days after the initiation of antifungal therapy, efforts should be made to identify any occult or persistent foci of candidemia.[^62^]

### Implementation of EBCS

The implementation of this EBCS is proposed with the aim of supporting strategies aimed at providing safe and quality care in institutions that provide health services to critically ill adults in the ICU in Latin America, to ensure the accurate diagnosis and treatment of candidemia in this population within the framework of the activities that are part of the programs for optimizing the use of antifungals.

For this, it is recommended to conduct the measurement of the indicators presented in Table 4, which are proposed as control points, for their measured, and mandatory reporting with the frequency that each institution considers pertinent based on its daily clinical practice.

**Table 4:** Indicators for the implementation of the EBCS.

Name	Definition	Numerator	Denominator
1. Sufficient sample collection for blood cultures	Proportion of patients with clinical suspicion of candidemia, with at least two sets of blood cultures taken (four bottles in total).	Total number of critically ill adult ICU patients with clinical suspicion of candidemia, with at least two sets of blood cultures taken (four bottles in total) of peripheral blood or blood drawn through the CVC.	Total number of critically ill adult ICU patients with clinical suspicion of candidemia
2. *Candida* genus and species identification.	Proportion of patients with isolation of *Candida* spp. in at least one blood culture bottle, with genus and species identification from the positive blood culture bottle.	Total number of critically ill adult ICU patients with isolation of *Candida* spp. in at least one blood culture bottle of peripheral blood or blood drawn through the CVC, with genus and species identification from the positive blood culture bottle.	Total number of critically ill adult ICU patients with isolation of *Candida* spp. in at least one blood culture bottle of peripheral blood or blood drawn through the CVC.
3. Use of an echinocandin as initial antifungal therapy.	Proportion of patients diagnosed with candidemia, with use of an echinocandin (caspofungin, micafungin, or anidulafungin) as initial antifungal therapy.	Total number of critically ill adult ICU patients with a diagnosis of candidemia, with use of an echinocandin (caspofungin, micafungin, or anidulafungin) as initial antifungal therapy.	Total number of critically ill adult ICU patients with a diagnosis of candidemia.
4. Removal of the central venous catheter	Proportion of patients diagnosed with candidemia, in whom the CVC is considered the source of the candidemia, with CVC removal as soon as possible.	Total number of critically ill adult ICU patients with a diagnosis of candidemia, in whom the CVC is considered the source of the candidemia, and it is possible to remove the CVC, with removal of the CVC as soon as possible.	Total number of critically ill adult ICU patients with a diagnosis of candidemia, in whom the CVC is considered the source of the candidemia, and the CVC can be removed.
5. Taking follow-up blood cultures.	Proportion of patients with a diagnosis of candidemia and antifungal treatment, with blood cultures taken 72 hours after the initiation of antifungal treatment.	Total number of critically ill adult ICU patients with a diagnosis of candidemia and antifungal treatment, with blood cultures taken 72 hours after the initiation of antifungal treatment.	Total number of critically ill adult ICU patients with a diagnosis of candidemia and initiation of antifungal treatment.
6. Transition to targeted therapy	Proportion of patients with a diagnosis of candidemia and antifungal treatment, with identification of a species other than *N. glabratus* (*C. glabrata*) or *C. auris*, susceptible to fluconazole or voriconazole, with transition to fluconazole or voriconazole.	Total number of critically ill adult ICU patients with a diagnosis of candidemia and initiation of antifungal treatment, with identification of a species other than *N. glabratus* (*C. glabrata*) or *C. auris*, susceptible to fluconazole or voriconazole, with transition to fluconazole or voriconazole.	Total number of critically ill adult ICU patients with a diagnosis of candidemia and antifungal treatment instituted, with identification of a species other than *N. glabratus* (*C. glabrata*) or *C. auris*, susceptible to fluconazole or voriconazole
7. Antifungal treatment duration	Proportion of patients with a diagnosis of candidemia and antifungal treatment, with a duration of antifungal therapy of 2 weeks, based on the documented elimination of *Candida* spp species. of the bloodstream and resolution of symptoms attributable to candidemia	Total number of critically ill adult ICU patients with a diagnosis of candidemia and antifungal treatment, with a duration of antifungal therapy of 2 weeks, based on documented elimination of *Candida* spp. of the bloodstream and resolution of symptoms attributable to candidemia	Total number of critically ill adult ICU patients with a diagnosis of candidemia and antifungal treatment, with documented elimination of *Candida* spp. of the bloodstream and resolution of symptoms attributable to candidemia.

To facilitate the implementation of this guide, the following dissemination tools will be used to facilitate its access to health professionals: the publication of the EBCS in the Brazilian Journal of Infectious Diseases; and the inclusion of the algorithms and recommendations as part of the contents of online courses and mobile applications.

### EBCS update

This EBCS should be updated within a maximum of five years, following the methodology and standards that have been established for developing evidence-based recommendations. The topics may be reconsidered according to the need for or publication of new evidence.

### Ethical considerations

The regulations outlined in national and international legislation have guided the development of this work, adhering to the ethical and bioethical standards for scientific research.

### Editorial independence

The content of this document was developed entirely independently, free from the influence of the funding entity, Pfizer Inc., which did not participate or have any role in the different phases of the development of the EBCS, including among others: the formation of the development group; the systematic search, screening, and selection of CPG; the analysis and interpretation of the evidence; the formation of the expert consensus; the generation of recommendations and preparation of the algorithms; the drafting of the manuscript; and the decision to publish. None of the professionals who were part of the development group, or the expert consensus received any type of payment or incentive for their participation in or contributions to the development of this document.

## Authors’ contributions

JAC conceptualized and led the EBCS. MCV performed the methodological coordination, systematic search of CPGs, and preparation of the comparative evidence table. JAC, MCV, CD, RRR, and LCN contributed to the screening, quality assessment, and selection of CPG, wrote the first draft of the manuscript and the final version. JAC, MCV, PMP, MSJ, CD, RRR, CJEA, and LCN participated as members of the development group in defining the scope and objective of the EBCS and in the process of reviewing, discussing, and drafting the proposed recommendations and preliminary algorithm. MN, CAA, FQ, RR, RR, JLF, FR, PC, DEC, LECP, JZ, ARH, and ALC participated as members of the expert consensus in the review, discussion, and final elaboration of recommendations and algorithms. The initial iteration of the manuscript was reviewed by all authors, and the final version was read and approved by all authors.

## Source of financing

This document has been developed as part of the “Antifungal administration programs in a Latin American country” project of the Universidad Nacional de Colombia, financed by a grant from Pfizer Inc. The project's objectives included developing recommendations for diagnosing and treating candidemia in critically ill ICU patients in Latin America.

## Conflicts of interest

The following authors declared that they have no conflict of interest: MSJ, CDB, RRR, CJEA, LCN. The following authors declared conflicts of interest: JAC (Pfizer), MCV (Pfizer), PMP (GlaxoSmithKline), MN (Mundipharma, Knight Therapeutics, Cidara Therapeutics), CAA (Moderna, Merck Sharp & Dohme, GlaxoSmithKline, Becton Dickinson, Pfizer), FQT (IMMY, Sandoz, Mundipharma), RR (Gilead Sciences, Gador, Pfizer), RRF (Pfizer, Gilead Sciences, Merck Sharp & Dohme), FR (Gilead Sciences, Janssen: Pharmaceutical Companies of Johnson & Johnson), PC (Pfizer, Merck Sharp & Dohme), DEC (IMMY), LECP (Knight Therapeutics), JZ (Pfizer), ARH (Octapharma, Pfizer, Medtronic), ALC (Grant from the Research Support Foundation of the State of São Paulo [FAPESP], number 2021/10,599–3), without identifying declared interests that could be considered potentially conflicting with the primary interest of this EBCS.

## Appendix Supplementary materials

Image, application 1
