# Supplementary material for: Evidence-based clinical standard for the diagnosis and treatment of candidemia in critically ill patients in the intensive care unit
Source: Braz J Infect Dis. 2024 Dec 21;29(1):104495. doi: 10.1016/j.bjid.2024.104495 (PMC11846572; doi:10.1016/j.bjid.2024.104495)
Supplement: Supplementary file 1 [file mmc1.docx]

**BJID-D-24-00206_ Supplementary material**

**Supplementary material**

**Table 1** Full search strategies for databases.

| **MEDLINE search strategy:** | |
| --- | --- |
| 1 | exp Diagnosis/ |
| 2 | diagnos*.ti,ab. |
| 3 | exp Therapeutics/ |
| 4 | (therap* or treatment* or manag*).ti,ab. |
| 5 | or/1-4 |
| 6 | exp Candida/ |
| 7 | exp Candidiasis/ |
| 8 | exp Candidiasis, Invasive/ |
| 9 | exp Candidemia/ |
| 10 | (Candid* adj2 (infecio* or fungemia* or disseminat* or systemic* or invasiv*)).ti,ab. |
| 11 | (Fung* adj2 (infecio* disseminat* or systemic* or invasiv*)).ti,ab. |
| 12 | Monilia*.ab,ti. |
| 13 | or/6-12 |
| 14 | exp Critical Illness/ |
| 15 | (Critical* adj2 Ill*).ab,ti. |
| 16 | exp Intensive Care Units/ |
| 17 | ICU.ab,ti. |
| 18 | or/14-17 |
| 19 | 5 and 13 and 18 |
| 20 | exp practice guidelines as topic/ |
| 21 | exp practice guideline/ |
| 22 | practice guideline.pt. |
| 23 | (practice adj2 guide*).ti,ab. |
| 24 | guideline.pt. |
| 25 | consensus development conference.pt. |
| 26 | recommendation*.ti,ab. |
| 27 | or/20-26 |
| 28 | 19 and 27 |
| 29 | limit 28 to yr="2014 -Current" |
| **EMBASE search strategy:** | |
| 1 | 'diagnosis'/exp |
| 2 | diagnos* |
| 3 | 'therapy'/exp |
| 4 | therap* OR treatment* OR manag* |
| 5 | OR/1-4 |
| 6 | 'candida'/exp |
| 7 | 'candidiasis'/exp |
| 8 | 'invasive candidiasis'/exp |
| 9 | 'candidemia'/exp |
| 10 | candid* NEAR/2 (infecio* OR fungemia* OR disseminat* OR systemic* OR invasiv*) |
| 11 | fung* NEAR/2 (infecio* OR disseminat* OR systemic* OR invasiv*) |
| 12 | 'monilia*' |
| 13 | OR/6-12 |
| 14 | 'critical illness'/exp |
| 15 | ('critical*' NEAR/2 'ill*'):ab,ti |
| 16 | 'intensive care unit'/exp |
| 17 | 'icu':ab,ti |
| 18 | OR/14-17 |
| 19 | #5 AND #13 AND #18 |
| 20 | 'practice guideline'/exp |
| 21 | practice* NEAR/2 guide* |
| 22 | guideline* |
| 23 | consensus NEAR/2 development NEAR/2 conference |
| 24 | recommendation* |
| 25 | OR/20-24 |
| 26 | #19 AND #25 |
| 27 | #26 AND (2014:py OR 2015:py OR 2016:py OR 2017:py OR 2018:py OR 2019:py OR 2020:py OR 2021:py OR 2022:py) |

**Figure 1** PRISMA flowchart.
